# Paired Ductal Carcinoma *In Situ* and Invasive Breast Cancer Lesions in the D-Loop of the Mitochondrial Genome Indicate a Cancerization Field Effect

**DOI:** 10.1155/2013/379438

**Published:** 2012-12-26

**Authors:** Andrea Maggrah, Kerry Robinson, Jennifer Creed, Roy Wittock, Ken Gehman, Teresa Gehman, Helen Brown, Andrew Harbottle, M. Kent Froberg, Daniel Klein, Brian Reguly, Ryan Parr

**Affiliations:** ^1^Mitomics Inc., 290 Munro Street, Suite 1000, Thunder Bay, ON, Canada P7A 7T1; ^2^Department of Surgery, Thunder Bay Regional Health Sciences Centre, 980 Oliver Road, Thunder Bay, ON, Canada P7B 6V4; ^3^Mitomics Inc., UK Ltd. Cels At Newcastle, Medical School, University of Newcastle, Framlington Place, Newcastle Upon Tyne NE2 4HH, UK; ^4^Department of Pathology, Saint Mary's Duluth Clinic, 400 East Third Street, Duluth, MN 55805, USA

## Abstract

Alterations in the mitochondrial genome have been chronicled in most solid tumors, including breast cancer. The intent of this paper is to compare and document somatic mitochondrial D-loop mutations in paired samples of ductal carcinoma *in situ* (DCIS) and invasive breast cancer (IBC) indicating a potential breast ductal epithelial cancerization field effect. Paired samples of these histopathologies were laser-captured microdissected (LCM) from biopsy, lumpectomy, and mastectomy tissues. Blood samples were collected as germplasm control references. For each patient, hypervariable region 1 (HV1) in the D-loop portion of the mitochondrial genome (mtGenome) was sequenced for all 3 clinical samples. Specific parallel somatic heteroplasmic alterations between these histopathologies, particularly at sites 16189, 16223, 16224, 16270, and 16291, suggest the presence of an epithelial, mitochondrial cancerization field effect. These results indicate that further characterization of the mutational pathway of DCIS and IBC may help establish the invasive potential of DCIS. Moreover, this paper indicates that biofluids with low cellularity, such as nipple aspirate fluid and/or ductal lavage, warrant further investigation as early and minimally invasive detection mediums of a cancerization field effect within breast tissue.

## 1. Introduction

In 2010, close to 207,090 new cases of IBC were diagnosed in the United States, and DCIS was identified in an additional 54,010 women who did not yet have IBC [1]. These statistics indicate that breast cancer and the often associated precursor lesion, DCIS, are global health problems.

Although DCIS masses are often small by comparison to IBC masses, DCIS is typically detected through mammography or self-examination. However, there are significant shortcomings to these methods. Mammography is generally used to identify breast masses within a resolution limit of 1 cm, and the Van Nuys prognostic index (VNPI) for DCIS does not score tumors less than 1.5 cm. This means that a subset of smaller lesions which may have significant future clinical impact remain undetected and/or evaluated. Breast self-examination has also been extensively shown to be an ineffective detection tool for Asian women [2, 3].

Currently, there is no clinical means to distinguish between the heterogeneous types of DCIS and recognize the carcinomas that will progress into invasive, metastatic breast cancer. The mechanism that drives this transformation from DCIS to IBC is also not well understood. Hence, when mammography detects DCIS, a full diagnostic workup and treatment is required [4]. As such, a huge need exists for the development of early detection procedures or tools for preinvasive lesions. If a link is established between smaller DCIS lesions and larger IBC lesions, or if a distinction can be made between invasive and noninvasive masses, it may then be possible to apply this knowledge to the development of early detection tools and chemopreventive treatment for women at risk.

The progression of DCIS is poorly understood because the technology used to detect it relies on tissue mass. Indeed, the identification of DCIS via mammography is low compared to larger tumors. If a significant proportion of IBC cases originate as DCIS, then successful detection and stratification of these lesions will assist the clinician and the patient with determining potential monitoring and treatment strategies. A recent review articulated the need for a combined research effort directed towards this clinical need [5].

This study proposes using somatic mitochondrial D-loop mutations in paired samples of DCIS and IBC to identify a potential breast ductal epithelial “cancerization” field effect. Alterations in the mitochondrial genome have been chronicled in most solid tumors, including breast cancer [6]. Since the mtGenome has an accelerated mutation rate in association with the beginning or presence of malignant transformation, patient-matched characterization of this genome in both DCIS and IBC may reveal a common related or progressive mutation pattern between these two lesions.

Mitochondrial D-loop mutations can be evaluated using tissue samples from solid tumors. Using biofluids with low cellularity such as nipple aspirate fluid (NAF) or ductal lavage (DL) represents a much less invasive route for developing early detection tests. The mtGenome is ideal for these investigations because it has a high copy number per cell, when compared to the nuclear archive of DNA.

There are other characteristics suggesting that the mtGenome may be an ideal “biosensor” as follows: (1) each copy of the mtGenome is clonal; (2) the mtGenome has a maternal inheritance pattern which precludes generational recombination; (3) somatic mutations appearing in a subset of mtGenomes, known as heteroplasmy, afford early disease detection; (4) the modest size of the mtGenome (16,568 bp) allows inexpensive, targeted, and concentrated genetic analyses; (5) the mtGenome has a 10–100-fold copy advantage over the nuclear genome; (6) the mitochondrial organelle is the center of ATP synthesis and is the mediator of cell apoptosis, and for successful tumorigenesis to occur, energy production must be replaced by an alternative process and apoptosis must be by-passed; (7) mitochondrial DNA (mtDNA) has an accelerated somatic mutation rate in which mutations occur within years, and perhaps months, from when molecular pathways are altered by early molecular changes associated with malignant transformation; (8) mutations in the mtGenome have been attested in a wide variety of solid tumors.

## 2. Materials and Methods

### 2.1. Patients and Samples

Women who were referred to a surgical oncologist for a clinical breast examination and had a biopsy with positive results were recruited to this study. Patients having a biopsy, lumpectomy, or mastectomy were selected based on a pathology report which identified both DCIS and IBC. Two patients had both a biopsy and a secondary procedure (lumpectomy and mastectomy). All patients were procured in accordance with the ethical guidelines of the Thunder Bay Regional Health Sciences Research Ethics Board in adherence to the Tri-Council Policy Statement on Ethical Conduct for Research Involving Humans. Written consent was obtained from the patients for publication of the study. Patients were selected based on review of biopsy and/or surgical pathology reports. A total of 34 patients were identified, however, upon sectioning of requested samples, only 15 had sufficient quantities of both IBC and DCIS to warrant LCM. After complete sample processing (extraction through sequencing), 5 patients were further eliminated due to sample drop-out. A total of 34 samples, including blood, were contributed by a suite of 10 patients (Table 1). Blood from a finger prick was collected on IsoCode cards (Whatman, Piscataway, NJ). DNA was extracted using a QIAcube (Qiagen, Germantown, MD) and QIAamp DNA mini kit (Qiagen, Germantown, MD) using the protocol for DNA purification for dried blood.

### 2.2. Laser Capture Microdissection

Requested tissues (biopsy and mastectomy samples) were sectioned from formalin-fixed paraffin-embedded (FFPE) blocks and processed for LCM. LCM was performed by two qualified, gowned, gloved, and masked technicians who captured both DCIS and IBC from each patient. By direct observation of the process, about 3-4 cells were harvested per laser pulse, or capture event, and approximately 2,000 captures were recovered from each tissue type. DNA was liberated from LCM samples by an overnight digestion at 65°C in 50 *μ*L of 100 mM Tris-HCl (pH 8.0), 10 mM EDTA, 1% Tween 20, and 20 mg/mL Proteinase K. The following morning, the reactions were inactivated at 95°C for 10 minutes. A total of 24 FFPE samples were processed. DNA was extracted using a QIAcube and QIAamp DNA mini kit tissue protocol, with the addition of heating each sample at 90°C for 1 hour after incubation of the sample at 56°C with 180 *μ*L of Buffer ATL plus 20 *μ*L Proteinase K. The samples were eluted in 200 *μ*L of Buffer AE. Samples were dried down and resuspended in 30 *μ*L of ddH_2_0.

### 2.3. Mitochondrial D-Loop Amplification

A portion of the D-loop was amplified with primer sets MT1, 2, 3 forward and reverse (MitoScreen Assay Kit, Transgenomic, Omaha, NE) using the following reagent concentrations per reaction: 1X FastStart High Fidelity Reaction Buffer, 1.8 mM MgCl_2_, and 0.25 U FastStart High Fidelity Enzyme Blend (Roche, Burgess Hill, UK); 0.2 mM of each dNTP; 0.3 *μ*M of each primer; 2.5 *μ*L of tissue extract or 1 *μ*L of DNA recovered from blood, with the final reaction volume adjusted to 25 *μ*L with ddH_2_0. Reactions were activated at 95°C for 6 minutes, then amplified with the following profile for 42 cycles: 95°C, 30 seconds; 56°C, 30 seconds; 72°C, 1 minute; followed by a final extension for 7 minutes at 72°C.

### 2.4. Denaturing High Performance Liquid Chromatography (DHPLC)

Following amplification, DHPLC was used to identify areas of mtGenome alterations. Sample analysis temperatures were predicted using Navigator software (Transgenomic, Omaha, NE). The gradient mobile phase consisted of Buffer A (0.1 M Triethylammonium Acetate pH 7.0) and Buffer B (0.1 M Triethylammonium Acetate pH 7.0) and 25% Acetonitrile. These buffers were mixed to provide a linear gradient varying Buffer B, for MT1 59–67, MT2 57–65, and MT3 59–67 over a 4-minute period. After each analysis, the column was cleaned with 100% Buffer B and then equilibrated for 2 minutes with Buffers A : B 54 : 56 (MT1 and MT3) 52 : 58 (MT2). Analysis temperatures were 58°C for MT1, 60°C for MT2, and 57°C and 59°C for MT3. Prior to injection, the samples were heteroduplexed by heating to 95°C for 5 minutes and then cooling at 1.5°C per minute down to 25°C.

### 2.5. Amplification of Altered Sequences Identified by DHPLC

Due to the low amount of template recovered from the LCM procedure, sequencing efforts were limited to a target sequence through and around hypervariable region 1 (HV1; 16,024–16,383). HV1 has a 2-fold higher mutation rate than HV2 [7]. A large fragment (1264 bp), including HV1, was amplified as previously mentioned with the following changes: primers MT2 and 19 from the MitoScreen Assay Kit (Transgenomic, Omaha, NE) concentrations were increased to 0.4 *μ*M, cycle number was reduced to 35, and the extension time was increased to 3 minutes.

This provided a low yield product which was amplified for a smaller sequence (627 bp) with nested primer D1 [8], which contains standard sequencing primer sites. The target sequence amplified by these primers specifically encompasses HV1 and flanking segments.

Reaction conditions were again the same as mentioned previously with the following changes: primer concentrations were increased to 0.6 *μ*M, and 1 *μ*L of the preceding product was used to seed the reaction which was run with the following conditions: 95°C for 6 minutes, 14 cycles of 94°C for 1 minute, 65°C for 1 minute (0.5°C per cycle), and 72°C for 1 minute. Then 20 cycles of 94°C for 1 minute, 58°C for 1 minute, 72°C for 1 minute, followed by a final extension of 72°C for 8 minutes.

### 2.6. Sequencing

Primer set MT2/MT19 (15424-102) was used to generate template for nested amplification with D1 primers (15898–16525). Both sets of primers were tested for null amplification against Rho 0 derived template [9] using the PCR conditions described previously to preclude the possibility of coamplification of numts. This mandatory precaution has been chronicled elsewhere [10, 11]. In addition, results were compared to the HV1 sequence signature of everyone directly involved with handling the samples to detect any incidental contamination by laboratory personnel. Finally, the corresponding germ plasma-derived DNA was amplified and sequenced from each patient as a direct comparative to control for actual somatic mutations as opposed to maternal variation.

Amplified template was sequenced at Genevision (Newcastle Upon Tyne, UK). Both Geneious bioinformatics software (Biomatters) and Sequencher 4.5 (Gene Codes) were used for sequence analyses.

### 2.7. Statistical Analyses

Analyses were performed on HV1 mutation patterns and all applicable parameters listed in the pathology report: age, receptor status, tumor grade, nuclear grade, tubule formation, mitotic score, modified Bloom-Richardson grade, and presence or absence of extensive intraductal component. Attempts to correlate the diagnostic rankings and per-site mutation results were made using point-biserial and rank-biserial statistics. Pearson rank correlation was used to identify the strength of the relationship between HVR1 relative substitution rates and the prevalence of each mutation site in the patient data. IBC and DCIS sample populations were considered separately in order to determine if any patterns existed in the mutation load of the individual sample types as well as to discover the presence of any interactions between the two tissue types. Again, Pearson correlations were used as statistics for this analysis.

## 3. Results

Mutations were identified in HV1 which was reamplified with Rho 0 null primers and sequenced. All patients in this study demonstrate heteroplasmy in all of the associated histologies in comparison to germ plasma, or blood. It is important to note that 18 sites had homoplasmic and/or heteroplasmic mutation sites in common between DCIS and IBC lesions recovered from the same patient. All patients had at least 1 corresponding homoplasmic and/or heteroplasmic site in both DCIS and IBC. These results parallel similar observations noting that other biomarkers are held in common between DCIS and IBC [12]. Two patients (43 and 74) had equivalent mutations in both biopsy samples and tissue from follow-up procedures (lumpectomy and mastectomy). See Tables 1 and 2 for an overview of clinical pathology and HV1 somatic mutations, respectively. No exogenous contamination from laboratory personnel, via comparison to HV1 sequence from germplasm, was observed.

### 3.1. Site Specific Mutations

Of the patients in this study, 9/10 had a mutation at positions 16189 and 16224 in at least one of their samples. Site 16189 may be of particular interest, as it is found in both DCIS and IBC in every patient. Mutations at position 16270 occur in 80% of the patients studied, but only about one third of these patients had this mutation in both histopathologies. 37.5% of the patients had this mutation in DCIS only, and 37.5% had this mutation in IBC exclusively. Three other sites, 16223, 16270, and 16291, occur in 8 patients, 2 sites (16311, 16362) in 7 patients, 1 site (16390) in 6 patients, 1 site (16304) in 4 patients, 1 site (16126) in 3 patients, and 2 sites (16093 and 16298) in 2 patients. Finally, 6 sites (16188, 16192, 16203, 16249, 16319, and 16357) are exclusive to 6 individual patients.

### 3.2. Clinical Correlation and Mutation Load

There appears to be no statistically significant correlation between single individual mutation sites and specific gradings; namely, the modified Bloom-Richardson grade, nuclear grade, tubule formation, and mitotic score.

The mutation loads of the IBC and DCIS samples were similar, even though up to a third of the mutations for a given patient differed. The average mutation load per patient was the same.

Considering IBC and DCIS mutation load from a per-site perspective, the two populations strongly correlate (*r* = 0.929, *P* < 0.001), meaning that the mutation load at a given site is consistent, regardless of tissue type. This may imply that the same damage is occurring in both tissues and that the disease processes may be similar.

## 4. Discussion

The observed frequency of mutations in the study population indicates a medium correlation with the relative mutation rates in HV1. All of the identified sites have estimated relative rates greater than zero, and 65% of the sites are classified as “fast” by multiple studies since they have a greater tendency to mutate than other neighboring sites. Using the same metric (substitution rate >2), 88% of the identified sites could be classified as “fast” [7].

The mutation sites identified by this study appear predisposed towards mutation. Since sites such as 16189 and 16224 are present in almost every patient, they demonstrate near confluence in this small cohort. This is perhaps due to a biological propensity to rapid mutation. As such, this attribute could be used as a breast cancer marker if this behavior is consistent in transforming breast tissue.

These results are consistent with a field effect demonstrated in epithelial tissues in general, including those cells lining the mammary ducts [13]. This field effect was also observed by Xu et al. in a small segment of the D-loop referred to as D310 [14]. This idea is demonstrated in multiple matching heteroplasmic and homoplasmic changes in HV1 in corresponding patient-matched DCIS and IBC samples from 10 patients in the study. A gland-wide influence is further suggested by the results of patients 43 and 74. Here, common mutations are observed in tissues from separate clinical procedures. Patient 43 has 3 mutations which occur in both biopsy and lumpectomy samples, in DCIS and IBC captured from biopsy and DCIS taken from a later lumpectomy. Patient 74 has 5 parallel alterations between IBC and DCIS from biopsy and DCIS recovered after a mastectomy. The IBC from mastectomy share 2 of these sites. This sample also has 2 unique changes.

Unfortunately, only patients 43 and 74 had follow-up procedures allowing this level of comparative analyses. The IBC and DCIS from the remaining 8 study participants were associated with 1 procedure, a biopsy, lumpectomy, or mastectomy. Absence of a 1 : 1 correlation between the mutation patterns of IBC and DCIS for a given patient and between separate procedures is likely a result of capturing ducts from tissue cross-sections and the convoluted anatomy of ductal tissue (i.e., patient 43). The extent and effect of the field may vary among associated, parallel ducts. Also, heteroplasmic signal detection up to 20% may not have been reached in all comparative patient samples.

Both telomere content (TC) and allelic imbalance (AI) have been documented in histologically normal breast tissue at 1 cm from a tumor focus. At 5 cm from a focus, TC and AI reflect normal parameters. This field could be much wider than 1 cm, since data was collected only at 1 and 5 cm intervals [15]. Similar epithelial field attributes have also been noted in lung cancer [16]. Also, extensive cancerization fields have been described in both head and neck cancers (7 cm in diameter) and colon cancer (3–10 cm in diameter) [17, 18]. The size of these fields may depend on the biological characteristics of the specific biomarkers.

It has been reported that D-loop mutations are associated with tumors which are both estrogen and progesterone receptor negative in women 50 years of age or older [18]. That pattern was not seen here which means that the D-loop alterations identified in this study would be suitable for use in a broad age range of women. Moreover, women with alterations in the D-loop experience poorer outcomes than those free of mutations [19]. This suggests that HV1 mutations found in both DCIS and IBC, when found in patients with DICS only, may be indicators of DCIS with potential aggressive behavior.

In other work, NAF was successfully retrieved from 82% of the participants with 96% yielding fluid from both breasts [20]. Given that the alterations displayed by the mtGenome demonstrate a field effect in breast tissue, there is merit in assessing NAF or DL recovered from women with both DCIS and IBC histopathologies. This applies to other abnormal breast histopathologies as well, such as atypical ductal hyperplasia. Both NAF and DL have been investigated as a source of biomarkers and for biological indications of breast cancer [16, 20–31]. Given the high copy number of the mtGenome and its rapid mutation rate, sequence analysis of the D-loop may identify mutations associated with these lesions in glandular organ-associated biofluids which are low in both volume and cellularity. Full mtGenome sequencing was successful for NAF and blood from 19 women referred to a surgical oncologist for a clinical breast examination and who had a nonmalignant outcome. A subset of these patients had a single mutation each (4/19, 21%) in the entire mtGenome. Unfortunately, no follow-up information was available for these women, and thus, comments regarding the association of the mutations observed with a disease state could not be reported.

## 5. Conclusions

This study was able to identify mtGenome alterations that occur in both DCIS and IBC within individual patients that are suggestive of a cancerization field effect, and DCIS that may be aggressive in nature. Other work demonstrates that large amounts of genetic information can be recovered from the high-copy-number mtGenome in low volume biofluids [20]. Identification of biomarkers with early detection and/or diagnostic capacity that utilize the mtGenome and its characteristics, in combination with the epithelial field effect and the use of NAF and/or DL as the detection medium, may have important clinical applications. Further studies are warranted to help unravel the mechanisms linking DCIS and IBC, as well as the mechanism that drives the transition from the smaller DCIS lesions to larger IBC lesions.

## Figures and Tables

**Table 1:** Clinical, pathological information with parallel mutation sites in common between both DCIS and IBC for each patient, inclusive of all patient tissue samples. Heteroplasmic sites are scored as mutations.

Patient	Age	Sample	Est	Pro	HER2	Grade	NG	TF	MS	BR	EIC	Parallel mutations	Haplotype
33	65	Mast	−	−	+	3	3	2	3	8	+	192, 224, 304, 311	U5a

36	67	Lump	+	−	−	1	1	1	1	3	−	188, 189, 223, 224, 270, 291, 311, 319, 362, 390	H

43	51	Biopsy					2					93, 189, 298	V
	52	Lump	+	+		2	2	2	2	6	−		

46	66	Biopsy	−	+	−	2	2	3	1	6	−	126, 189, 223, 291, 357, 362, 390	J

57	41	Lump	+	+	−	3	2	3	3	8	+	189, 291, 390	H

64	51	Biopsy										182, 183, 249	H
	51	Mast	−	−	+	3	3	3	3	9	−		

74	39	Biopsy	−	−	+							203, 304	K
	40	Mast				2	3	2	1	6	+		

83	49	Lump	+	−	−	2	2	3	1	6	−	189	H

89	41	Lump	−	−	−	3	3	3	3	9	−	189, 223, 224, 291, 298, 311, 362, 390	K

94	50	Lump	+	+		1	2	2	1	5	−	93, 189, 224, 291, 311	H

Unavailable information is left blank. NG: nuclear grade; MS: mitotic score; BR: modified Bloom-Richardson grade; EIC: extensive intraductal component.

**Table 2:** HV1 somatic mutations are bolded, while mutations persisting in all patient samples are also italicized. Patient histologies are compared to the corresponding sequence of their germplasm or blood (B) to detect mutations. Only those sites appearing in all histologies for a given patient are identified.

	93	126	188	189	192	203	223	224	249	270	291	298	304	311	319	357	362	390
RCRS	T	T	C	T	C	A	C	T	T	C	C	T	T	T	G	T	T	G

33 B	T	T	C	T	T	A	C	T	T	T	C	T	C	T	G	T	T	G
33 MIBC	N	N	C	T	***C ***	A	C	***C ***	T	T	C	T	***T ***	***C ***	G	T	T	G
33 MDCIS	T	**Y**	C	T	***C ***	A	C	***Y ***	T	**Y**	C	T	***Y ***	***Y ***	G	T	T	G

36 B	T	T	T	T	C	A	C	T	T	C	C	T	T	T	A	T	T	G
36 LIBC	T	T	***C ***	***Y ***	C	A	***Y ***	***Y ***	T	***Y ***	***Y ***	T	T	***Y ***	***G ***	T	***Y ***	***R ***
36 LDCIS	T	T	***C ***	***Y ***	C	A	***Y ***	***Y ***	T	***T ***	***Y ***	T	T	***Y ***	***G ***	T	***Y ***	***R ***

43 B	C	T	C	T	C	A	C	T	T	C	C	C	T	T	G	T	T	G
43 BIBC	***T ***	T	C	***Y ***	C	A	C	**Y**	T	**Y**	C	***T ***	T	T	G	T	T	G
43 BDCIS	***T ***	T	C	***Y ***	C	A	C	**Y**	T	C	C	***T ***	T	T	G	T	T	G
43 LDCIS	***T ***	**C**	C	***Y ***	C	A	**Y**	T	T	C	**Y**	***T ***	**Y**	T	G	T	**Y**	G

46 B	T	C	C	T	C	A	C	T	T	C	C	T	T	T	G	C	T	G
46 BIBC	T	***T ***	C	***Y ***	C	A	***Y ***	T	T	C	***Y ***	T	T	T	G	***T ***	***Y ***	***R ***
46 BDCIS	T	***T ***	C	***C ***	C	A	***T ***	T	T	C	***T ***	T	T	T	G	***T ***	***C ***	***A ***

57 B	T	T	C	***T ***	C	A	C	T	T	C	C	T	T	T	G	T	T	G
57 LIBC	N	N	C	***Y ***	C	A	**T**	T	T	C	***T ***	T	T	T	G	T	**C**	***A ***
57 LDCIS	T	T	C	***Y ***	C	A	C	**Y**	T	**Y**	***Y ***	T	T	Y	G	T	T	***R ***

64 B	T	T	C	C	C	A	C	T	C	C	C	T	T	T	G	T	T	G
64 MIBC1	N	N	C	C	C	A	**Y**	**Y**	***Y ***	**Y**	**Y**	T	T	**Y**	G	T	**Y**	**R**
64 MIBC2	T	T	C	**Y**	C	A	**Y**	T	***T ***	C	**Y**	T	T	T	G	T	**Y**	**R**
64 MDCIS	T	T	C	**Y**	C	A	C	**Y**	***Y ***	**Y**	C	T	T	**Y**	G	T	T	G

74 B	T	T	C	T	C	G	C	T	T	C	C	T	C	T	G	T	T	G
74 BIBC	T	T	C	**Y**	C	***A ***	**Y**	T	T	C	**Y**	T	***T ***	T	G	T	**Y**	**R**
74 BDCIS	T	T	C	**Y**	C	***A ***	**T**	T	T	C	**Y**	T	***T ***	T	G	T	**Y**	**R**
74 MIBC	T	T	C	T	C	***A ***	C	**Y**	T	**Y**	C	T	***T ***	T	G	T	T	G
74 MDCIS	T	T	C	**Y**	C	***A ***	**Y**	T	T	C	**Y**	T	***T ***	T	G	T	**Y**	**R**

83 B	T	T	C	C	C	A	C	T	T	C	C	T	T	T	G	T	T	G
83 BIBC	T	T	C	***T ***	C	A	**Y**	T	T	C	C	T	T	T	G	T	T	G
83 BDCIS	T	T	C	***T ***	C	A	C	**C**	T	C	C	T	T	**C**	G	T	T	G

89 B	T	T	C	T	C	A	C	C	T	C	C	C	T	C	G	T	T	G
89 BIBC	N	N	C	***Y ***	C	A	***Y ***	***T ***	T	C	***Y ***	***T ***	T	***T ***	G	T	***Y ***	***R ***
89 BDCIS	N	N	C	***Y ***	C	A	***Y ***	***Y ***	T	**Y**	***Y ***	***T ***	**Y**	***Y ***	G	T	***Y ***	***R ***

94 B	C	T	C	C	C	A	C	T	T	T	T	T	T	T	G	T	T	G
94 BIBC	***T ***	T	C	***T ***	C	A	C	***Y ***	T	**C**	***C ***	T	T	***Y ***	G	T	T	G
94 BDCIS	***T ***	T	C	***T ***	C	A	C	***C ***	T	T	***C ***	T	T	***C ***	G	T	T	G

IBC: invasive breast carcinoma, DCIS: ductal carcinoma *in* *situ*. Prefixes: B: biopsy, L: lumpectomy, and M: mastectomy. The revised Cambridge reference sequence (RCRS) is used as a standard comparison. The first 2 and/or 3 digits for each site have been removed to avoid redundancy.
